# Research on Trajectory Planning of Autonomous Vehicles in Constrained Spaces

**DOI:** 10.3390/s24175746

**Published:** 2024-09-04

**Authors:** Yunlong Li, Gang Li, Xizheng Wang

**Affiliations:** School of Automobile and Traffic Engineering, Liaoning University of Technology, Jinzhou 121001, China; 221285021@stu.lnut.edu.cn (Y.L.); 231285063@stu.lnut.edu.cn (X.W.)

**Keywords:** trajectory planning, hybrid A-star, quadratic programming, speed planning

## Abstract

This paper addresses the challenge of trajectory planning for autonomous vehicles operating in complex, constrained environments. The proposed method enhances the hybrid A-star algorithm through back-end optimization. An adaptive node expansion strategy is introduced to handle varying environmental complexities. By integrating Dijkstra’s shortest path search, the method improves direction selection and refines the estimated cost function. Utilizing the characteristics of hybrid A-star path planning, a quadratic programming approach with designed constraints smooths discrete path points. This results in a smoothed trajectory that supports speed planning using S-curve profiles. Both simulation and experimental results demonstrate that the improved hybrid A-star search significantly boosts efficiency. The trajectory shows continuous and smooth transitions in heading angle and speed, leading to notable improvements in trajectory planning efficiency and overall comfort for autonomous vehicles in challenging environments.

## 1. Introduction

The rapid development in autonomous driving technology has provided many conveniences to people’s lives and reduced traffic accidents [1]. Among them, path planning is one of the core research areas of autonomous driving technology, aiming to plan a path for the vehicle to reach the destination safely and ensure the avoidance of obstacles [2]. Although autonomous driving technology has been commercialized in some scenarios, it still faces greater challenges in path planning. In particular, there are avoidance of high-density obstacles under restricted and narrow working conditions, precise kinematic constraint handling, and real-time requirements. Path planning methods can be classified according to different technical means, including graph search-based methods, optimization methods, sampling-based methods, and machine learning methods. Among them, Dijkstra’s algorithm [3] and A-star algorithms [4,5], as the classical algorithms of a graph search, need to preform a path search on a discrete map to find the optimal path. Optimization methods utilize mathematical optimization techniques [6,7], such as linear programming and nonlinear programming [8], that are capable of handling complex dynamic environments and multi-objective constraints. Sampling-based methods such as RRT (Rapid Exploration Random Tree) and its variants [9,10] generate path candidates by random sampling and filter the optimal paths to be suitable for high-dimensional spaces and complex obstacle environments.

Dmitri Dolgov first proposed the hybrid A-star method, Sebastian Thrun et al., at Stanford University in 2008 [11]. Hybrid A-star combines the A-star algorithm, which “takes obstacles into account without considering motion constraints”, with the Reeds–Shepp curve, which “takes obstacles into account without considering motion constraints”. The hybrid A-star combines the A-star algorithm “considering obstacles without motion constraints” with the Reeds–Shepp curve [12] “considering motion constraints without obstacles”. Therefore, compared with other algorithms, the hybrid A-star is more suitable for trajectory planning at low speeds in restricted spaces. After years of development, hybrid A-star is improved to be applied in different working scenarios. Meng et al. [13] enhance the performance of hybrid A-star algorithms through a safety-enhanced design and an efficiency-enhanced design. The safety-enhanced design integrates the Voronoi field potential function in the path search phase to better consider the safety of the path. The efficiency-enhanced design proposes a multi-stage dynamic optimization strategy that divides the path planning into multiple stages and performs dynamic optimization at each stage. The problem that the output paths of the hybrid A-star algorithm often contain unnecessary steering maneuvers, and the paths are close to obstacles, is addressed. Tang et al. [14] propose a method that applies the concept of an artificial potential field to optimize the hybrid A-star algorithm. The generated paths not only satisfy the vehicle’s non-integrity constraints, but also smooth and maintain a comfortable distance from obstacles. Tian et al. [15] proposed a hybrid A-star path planning method based on hierarchical clustering and trilateration to solve the problems of poor path smoothness and long paths of self-driving cars in narrow areas. The method uses the Prewitt operator to identify obstacle boundaries and discretize them; a single-link hierarchical clustering algorithm is used for obstacle clustering; a convex packet algorithm is used to envelop the clustered points and extend the car to solve the problem of a traditional hybrid A-star algorithm’s extension in U-shape obstacle clusters; and, finally, the node extension strategy is improved based on the method of trichotomies. Jing et al. [16] proposed an enhanced hybrid A-star (EHA) algorithm to solve the problem of high computational cost or inability to find a suitable initial guess in narrow and complex environments. The EHA consists of four steps: first, the global rough trajectory is quickly obtained using traditional A; then, the driving corridor is constructed along the rough trajectory, and each channel node is evaluated; then, the channel boundary points are extracted; and finally, the boundary points are connected by hybrid A that generates a feasible initial guess for the OCP (Optimal Control Problem). Dang et al. [17] improved the RS method in the hybrid A-star algorithm by providing multiple curvature choices to improve safety and introducing a cost function that evaluates the risk of collision and the cost of motion. In addition, by fine-tuning the motion primitives in the forward search phase, unnecessary turning points are reduced, resulting in smoother paths.

Numerous scholars mentioned above pin the hybrid A-star algorithm with different improvements, and there exists a significant improvement in path smoothing, search efficiency, and obstacle avoidance ability. However, for actual tracking control of its planned path, it is necessary to assign desired vehicle speed and acceleration to each discrete path point, i.e., speed planning. Therefore, this paper proposes a method to improve the search efficiency of the hybrid A-star algorithm while performing back-end processing on its planned trajectories. Smoothing of the hybrid A-star planned trajectories and mapping the planned speeds to discrete path points are realized. An optimal trajectory containing position, heading, speed, and acceleration is planned. The main major contributions and innovations of this paper are as follows:

- Pre-processing using Dijkstra’s algorithm searches for the shortest path between the start and endpoints that can avoid obstacles. The hybrid A-star searches the path by calculating the estimated cost h(*n*) based on this shortest path to provide the correct direction guidance for the search. In addition, the node expansion strategy with variable step length and variable angle is designed according to the environment complexity and path completion.
- Consider the smoothing cost, discrete path point compact cost, and path geometric similarity cost to construct the quadratic programming problem. By designing constraints to ensure the bit position continuity at the articulation of the forward and reverse segments, the segmental smoothing of the planned path is realized.
- Design the speed planner according to the S-curve to carry out speed planning for the smoothed path, according to the characteristics of the path design speed planner adaptive system parameters’ adaptive strategy.

This paper consists of the following main sections in addition to this section: the second section describes the improvement in the hybrid A-star algorithm; the third section performs the back-end smoothing of the planning path; the fourth section is the velocity planning of the planning path; the fifth section validates the method proposed in this paper through simulations and experiments; and the last section gives the conclusions.

## 2. Improvement in the Hybrid A-Star Algorithm

**Figure 1:**
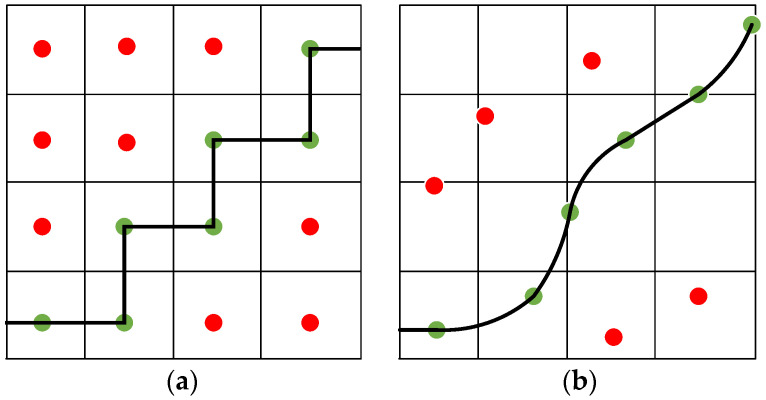
Comparison of different expansion methods; they should be listed as (**a**) the A-star extension method; (**b**) the hybrid A-star extension method.

Unlike the traditional search class algorithms Dijkstra and A-star algorithms, the hybrid A algorithm will consider the vehicle’s kinematic model constraints when performing the node expansion, which extends the two-dimensional planar search to the three-dimensional space [x, y, yaw], where yaw denotes the vehicle’s heading information, as shown in Figure 1. Hybrid A is able to plan the continuous positional changes in the vehicle in a discrete grid, making it practical and accurate in restricted spaces and narrow environments. In order to improve the utility of the hybrid A-star algorithm in path planning, this paper improves both the node expansion method and the cost function calculation method based on the traditional hybrid A-star algorithm to improve search efficiency and path quality, where the path quality mainly refers to the path length and curvature change.

### 2.1. Improving Node Expansion

In terms of node expansion methods, short-distance mobility has better flexibility and obstacle-bypassing ability, but it leads to an increase in the number of node expansions. While long-distance mobility has poor obstacle avoidance ability, it can approach the target point faster and reduce the number of node expansions. In this paper, we redesign the node expansion strategy by introducing the concepts of environmental complexity and planning completion. The advantages of short- and long-distance mobility methods are combined.

**Figure 2:**
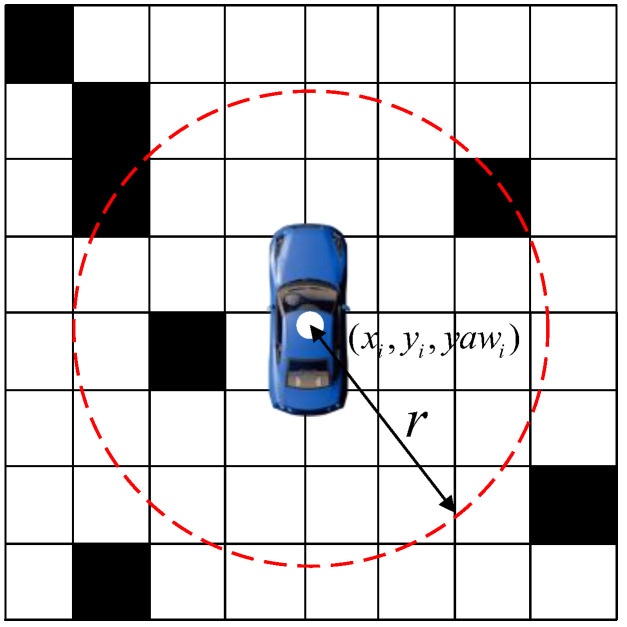
Environmental complexity schematics.

As shown in Figure 2 for the vehicle at the current node, set the current node as $\left(x_{i},y_{i},yaw_{i}\right)$. Traverse the raster range as a circle centered at the current node with radius r. Calculate the percentage of the circle occupied by obstacles $P_{obs}$:

(1) $$P_{obs}=\frac{N_{obs}}{N_{total}}$$

where $N_{total}$ is the total number of grids in the circle; and $N_{obs}$ is the number occupied by obstacles; for each grid, whose centroid coordinates are $\left(x_{grid},y_{grid}\right)$ inside the circle, by calculating the distance from the center of the grid to the center of the circle $d_{center}$,

(2) $$d_{center}=\sqrt{{\left(x_{grid}-x_{i}\right)}^{2}+{\left(y_{grid}-y_{i}\right)}^{2}}$$

If $d_{center}<r$, the raster is determined to be inside the circle. Define the function $C(x_{i},y_{i})$ to describe the environment complexity. The complexity of the environment refers to the proportion of occupied rosters around the current node to the total number of rasters.

(3) $$C(x_{i},y_{i})=w_{obs}P_{obs}$$

where $\omega_{obs}$ is the environmental complexity factor, and the Euclidean distance from the current node to the endpoint is $d_{i}$. Then, according to the environmental complexity and the planning progress, the dynamic extended distance step is defined as

(4) $$\Delta s_{i}=\min\left\{\Delta s_{0}*\frac{1}{1+\alpha C(x_{i},y_{i})}*(1+\beta \frac{d_{i}}{L_{i}+\epsilon }),\Delta s_{\max}\right\}$$

where $\Delta s_{0}$ is the base search step; $\Delta s_{\max}$ is the maximum search angle step; α and β are adjustment coefficients used to balance the environment complexity and path planning progress; $L_{i}$ is the Euclidean distance from the current node to the starting point; and ϵ is a small positive constant to avoid an infinite situation. Similarly, for the dynamic extended angle step,

(5) $$\Delta \theta_{i}=\min\left\{\Delta \theta_{0}*\frac{1}{1+\alpha C(x_{i},y_{i})}*(1+\beta \frac{d_{i}}{L_{i}+\epsilon }),\Delta \theta_{\max}\right\}$$

where $\Delta \theta_{0}$ is the base search angle step; $\Delta \theta_{\max}$ is the maximum search angle step.

When searching in three-dimensional space, the step length decreases when the complexity of the environment increases, ensuring a more detailed search in complex environments; when the distance from the target point is far, the step length is relatively large. To quickly approach the target point, and as the distance to the target point decreases, the step length gradually decreases to improve the accuracy of the path. Equations (4) and (5) demonstrate that when the distance to the target point is larger, and the environment is less complex, the step size of node expansion becomes more larger. To prevent overlooking small obstacles due to the large step size, we set upper limits for the search step sizes, $\Delta s_{max}$ and $\Delta \theta_{max}$. These upper limits are adjusted based on the size of the vehicle.

**Remark 1.**  *The choice of parameters regarding the adjustment coefficients α and β is related to the degree of conservatism of the algorithm. A more significant α means that the complexity of the environment is scaled up accordingly, and the step size of the expansion becomes relatively more minor. The larger β means amplifying the distance from the current node to the endpoint, and the step size of the expansion becomes relatively more significant. These two coefficients constrain each other, and the algorithm’s performance can be improved by choosing appropriate parameters.*

### 2.2. Heuristic Improvements

For search class algorithms, the heuristic function plays a crucial role in (1) guiding the search direction, (2) improving the search efficiency, (3) reducing unnecessary extensions, etc. Dijkstra’s algorithm, the classical method of search class planning, searches for paths based on the actual cost g(*n*) from the starting point to the current node, so the search scope is wider and less efficient. At the same time, the A-star algorithm combines the actual cost g(*n*) and the estimated cost h(*n*) and guides the search direction by a heuristic function so as to improve the search efficiency. The heuristic for the hybrid A-star algorithm, combined with the heuristic for the A-star algorithm, is designed as f(*n*) = g(*n*) + h(*n*). In this formulation, g(*n*) represents the actual cost, incorporating penalties for the length to the parent node, steering, steering changes, and reversals. The estimation of the cost h(*n*) includes the Manhattan distance from the current node to the target node and the length of the Reeds–Shepp curve [18] connection between the current node and the target node. Both of these penalties ignore the presence of obstacles. In complex environments (e.g., regions with dense obstacles or complex terrain), these simple heuristics do not accurately reflect the actual optimal path. This leads to a decrease in search efficiency or the generation of suboptimal paths. In order to improve the search efficiency and planning accuracy of the hybrid A-star algorithm in a narrow space, the estimation cost h(*n*) in it is improved:

(6) $$h(n)=w_{m}d_{man}+w_{n}d_{nearst}+w_{c}d_{curve}$$

where $d_{man}$ is the Manhattan distance from the pre-point node to the target node, defining the end position as $\left(x_{goal},y_{goal},yaw_{goal}\right)$:

(7) $$d_{man}=|x_{i}-x_{goal}|+|y_{i}-y_{goal}|$$

**Figure 3:**
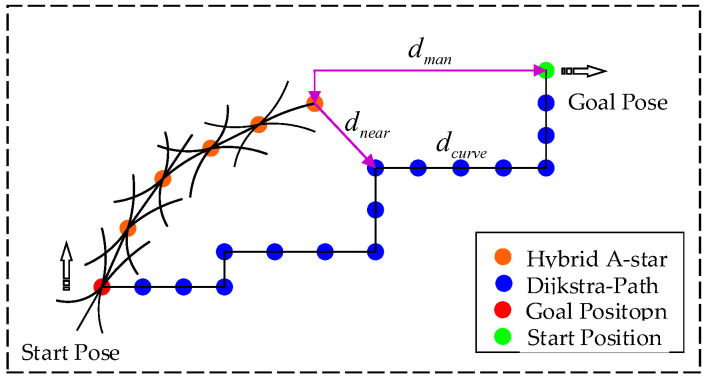
Schematic representation of estimated costs.

Distance from the current node to the nearest discrete point in the shortest path $d_{near}$:

(8) $$d_{near}=\min_{p\in dijkstra\_path}(\sqrt{{\left(x_{i}-x_{goal}\right)}^{2}+{\left(y_{i}-y_{goal}\right)}^{2}})$$

where dijstra_path is the set of discrete points in the shortest path pre-searched by Dijkstra. The length of the curve from the nearest discrete point to the target node $d_{curve}$:

(9) $$d_{curve}=\mathrm{Dijkstra}\text{-}\mathrm{Path}(p_{nearest},p_{goal})$$

where $p_{nearest}$ is the closest discrete point to the current node in the shortest path, as shown in Figure 3, being a schematic diagram of each of the h(*n*):

To ensure the planned path is optimal while meeting the vehicle’s kinematic characteristics and environmental constraints, a Reeds-Shepp curve is used to connect to the target point when the node expansion is close to the target, generating a smooth and feasible path. However, if the shape of the Reeds–Shepp curve at the end of the path is not considered when mixing the paths searched by the A-star, the curve at the end of the path will appear as a path with frequent reversals. The final design estimated cost h(*n*) is

(10) $$h(n)=\left\{\begin{array}{l} w_{m}d_{Man}+w_{n}d_{nearst}+w_{c}d_{curve}\;d_{goal}>shootdist\\ length_{reeds-shepp}+number_{rs-reverse}otherwise \end{array}\right.$$

## 3. Path Smoothing

**Figure 4:**
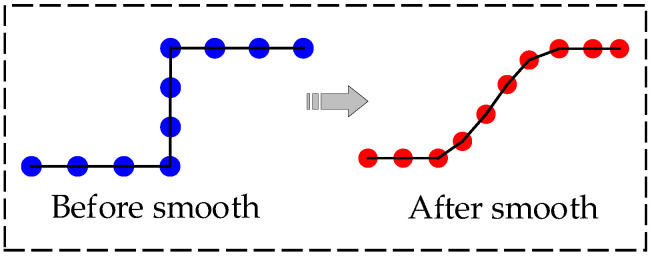
Schematic diagram of path smoothing process.

In the search process of the hybrid A-star algorithm, penalizing changes in vehicle heading and steering can ensure that the planned path minimizes the number of turns as much as possible. However, when turning is necessary, the vehicle will rotate at a high yaw rate. This reduces ride comfort and impacts the actuator. To address the above problems, back-end optimization of the paths planned by the improved hybrid A-star is performed. The steering maneuver of the vehicle is made smooth and continuous, as shown in Figure 4. The quadratic optimization problem is constructed through the geometric relationship between the path points, and the constraints are set according to the path characteristics to achieve the smoothing of the path.

### 3.1. Constructing a Cost Function

**Figure 5:**
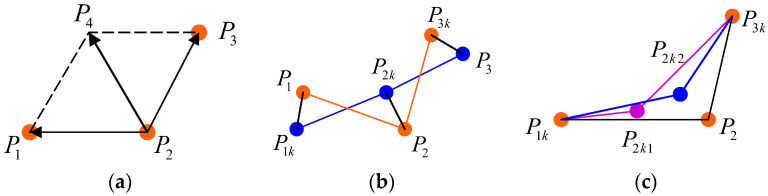
Schematic representation of costs; they should be listed as (**a**) schematic of smoothing cost of path points; (**b**) schematic diagram of compact cost of path points; (**c**) schematic of geometric similarity cost of paths.

The degree of smoothing after smoothing the path, the degree of compactness of the uniform distribution, and the degree of geometric similarity are considered for the establishment of the cost function, respectively. Figure 5 shows a schematic representation of the various costs.

In graph (a) of Figure 5, points $P_{1}$, $P_{2}$, and $P_{3}$ are three consecutive discrete points on the planning path, where $P_{4}$ is the vertex of the sum of vector $\left|P_{1}P_{2}\right|$ and vector $\left|P_{2}P_{3}\right|$. The length of vector $\left|P_{2}P_{4}\right|$ is used as a measure of path smoothing. In graph (b) of Figure 5, $P_{1}$, $P_{2}$, and $P_{3}$ are three discrete points on the continuous path; $P_{1k}$, $P_{2k}$, and $P_{3k}$ are the discrete points after smoothing. Although the smoothed path is a smooth, straight line, the deviation of the smoothed path from the original path is too large, which leads to planning failure. Therefore, the sum of vectors $\left|p_{1}p_{1k}\right|$, $\left|P_{2}P_{2k}\right|$, and $\left|P_{3}P_{3k}\right|$ is chosen as a measure of the geometric similarity of the paths. In graph (c) of Figure 5, $P_{1k}$ and $P_{3k}$ are the two endpoints after smoothing. $P_{2}$ is the intermediate discrete point before smoothing, and $P_{2k1}$ and $P_{2k2}$ are intermediate discrete points after smoothing in two cases. The planned path requires the discrete points to be uniformly distributed, and since the hybrid A-star algorithm searches with a fixed step size, the planned discrete points are uniformly distributed. Therefore, in the optimization, we need to ensure that the smoothed path points are uniformly distributed and compact. When point $P_{2}$ is point $P_{2k1}$ after smoothing, it satisfies $\left|P_{1k}P_{2k1}\right|>\left|P_{2k1}P_{3k}\right|$, and the three points after smoothing are not uniformly distributed. When point $P_{2}$ is smoothed to point $P_{2k2}$, it satisfies $\left|P_{1k}P_{2k2}\right|\approx \left|P_{2k2}P_{3k}\right|$, and the distribution of the three discrete points after smoothing is uniform, which meets the planning requirements. $|P_{1k}P_{2k}{|}^{2}+|P_{2k}P_{3k}{|}^{2}$ is selected as the evaluation index of uniform and compact distribution of path points after smoothing.

To summarize, the cost of the whole path smoothing is mainly composed of three parts: the smoothing cost, the path geometric similarity cost, and the compactness cost, which are expressed as follows:

(11) $$f_{1}={\left(x_{1}+x_{3}-2x_{2}\right)}^{2}+{\left(y_{1}+y_{3}-2y_{2}\right)}^{2}$$

(12) $$f_{2}=\sum_{i=1}^{3}{\left(x_{i}-x_{ki}\right)}^{2}+{\left(y_{i}-y_{ki}\right)}^{2}$$

(13) $$f_{3}=\sum_{i=1}^{3}{\left(x_{i+1}-x_{i}\right)}^{2}+{\left(y_{i+1}-y_{i}\right)}^{2}$$

where $x_{ki}$ and $y_{ki}$, i = 1, 2, and 3, are the known original discrete path points; $x_{ki}$ and $y_{ki}$, i = 1, 2, and 3, are the unknown smoothed path points; $f_{1}$ is the path smoothing cost function; $f_{2}$ is the geometrically similar cost to the original path points; and $f_{3}$ is the compact cost of the discrete path points. The total smoothing cost is designed as follows:

(14) $$cost=\omega_{1}f_{1}+\omega_{2}f_{2}+\omega_{3}f_{3}$$

where $\omega_{1}$ is the smoothing cost weight coefficient; $\omega_{2}$ is the cost of geometric similarity to the original path points; and $\omega_{3}$ is the cost of compactness of the path discrete points.

### 3.2. Constructing the Quadratic Programming Problem

The smoothed path discrete points need to satisfy the solution when the total cost of smoothing is minimized. Combining the characteristics of Equations (11)–(14), a method of constructing a quadratic programming problem for a solution is adopted. The standard form of quadratic programming [19] is given below:

(15) $$\begin{array}{l} \min\mathrm{imize}\;\frac{1}{2}x^{T}Hx+c^{T}x\\ \mathrm{subject}\;\mathrm{to}\;Ax\leq b\\ \;\;\;\;\;Ex=d \end{array}$$

where x is the optimization variable; H is the Hession matrix; c is the gradient vector; and s.t. is the equation and inequality constraints. Equation (14) is deformed according to its standard form, and the constraints are designed according to the actual needs.

#### 3.2.1. Smoothing Cost

For smoothing of three discrete path points, expanding Equation (11) yields

(16) $$f_{1}=(x_{1}+x_{3}-2x_{1},y_{1}+y_{3}-2y_{1}){\left(x_{1}+x_{3}-2x_{1},y_{1}+y_{3}-2y_{1}\right)}^{T}$$

where $(x_{1}+x_{3}-2x_{1},y_{1}+y_{3}-2y_{1})=(x_{1},y_{1},x_{2},y_{2},x_{3},y_{3})\left(\begin{array}{cc} 1 & 0\\ 0 & 1\\ -2 & 0\\ 0 & -2\\ 1 & 0\\ 0 & 1 \end{array}\right)$. Denote $x=\left(\begin{array}{l} x_{1}\\ y_{1}\\ x_{2}\\ y_{2}\\ x_{3}\\ y_{3} \end{array}\right)$ as the optimization variable. Then, Equation (11) can be rewritten as

(17) $$f_{1}=x_{1}^{T}A_{1}^{T}A_{1}x$$

When considering the case of n discrete path points, the smoothing cost $f_{1}$ is as follows:

(18) $$f_{1}=\sum_{i=1}^{n-2}{\left(x_{i}+x_{i+2}-2x_{i+1}\right)}^{2}+{\left(y_{i}+y_{i+2}-2y_{i+1}\right)}^{2}$$

Similarly, Equation (18) can be organized into the form of Equation (17), while x is a 1×2n matrix containing the horizontal and vertical coordinates of n discrete points. $A_{1}$ is a (2n−4)×2 matrix containing n−2 matrices.

#### 3.2.2. Compact Cost

Extending Equation (12) to the case of *n* discrete points, the process rounding cost of path smoothing can be written as

(19) $$f_{2}=\sum_{i=1}^{n-1}{\left(x_{i}-x_{i+1}\right)}^{2}+{\left(y_{i}-y_{i+1}\right)}^{2}$$

(20) $$f_{1}=(x_{1}-x_{2},y_{1}-y_{1},x_{2}-x_{3},y_{2}-y_{3},\ldots ){\left(x_{1}-x_{2},y_{1}-y_{1},x_{2}-x_{3},y_{2}-y_{3},\ldots \right)}^{T}$$

Let $A_{2}=\left(\begin{array}{cccccc} 1 & 0 & 0 & 0 & \;..... & \\ 0 & 1 & 0 & 0 & & \\ -1 & 0 & 1 & 0 & & \\ 0 & -1 & 0 & 1 & ..... & \\ 0 & 0 & -1 & 0 & & \\ 0 & 0 & 0 & -1 & & \\ 0 & 0 & 0 & 0 & & \\ & & ..... & & \;..... & \end{array}\right)$ and its dimension size be 2n×(2n−2). Finally, the compact cost can be organized as follows:

(21) $$f_{2}=x^{T}A_{2}^{T}A_{2}x$$

#### 3.2.3. Geometric Similarity Cost

For *n* discrete path points, the geometric similarity cost can be written as

(22) $$f_{3}=\sum_{i=1}^{n}{\left(x_{i}-x_{ki}\right)}^{2}+{\left(y_{i}-y_{ki}\right)}^{2}$$

Expanding Equation (22) yields

(23) $$f_{3}=\sum_{i=1}^{n}\left(x_{i}^{2}+y_{i}^{2}\right)+\sum_{i=1}^{n}\left(-2x_{ki}x_{i}-2y_{ki}y_{i}\right)+\sum_{i=1}^{n}\left(x_{ki}^{2}+y_{ki}^{2}\right)$$

Since $x_{ki}$ and $y_{ki}$ are known information, the third term in Equation (11) is constant, and hence the cost is not affected by it. Now, Equation (11) is converted into vector form as follows:

(24) $$f_{3}=(x_{1},y_{1},x_{2},y_{2},\ldots )\left(\begin{array}{cccc} 1 & 0 & 0 & \\ 0 & 1 & 0 & \\ 0 & 0 & 1 & \;\ldots \\ 0 & 0 & 0 & \\ & \ldots & & \end{array}\right)\left(\begin{array}{c} x_{1}\\ y_{1}\\ \ldots \\ \ldots \\ x_{n}\\ y_{n} \end{array}\right)+(-2)(x_{k1},y_{k1},x_{k2},y_{k2},\ldots )\left(\begin{array}{c} x_{1}\\ y_{1}\\ \ldots \\ \ldots \\ x_{n}\\ y_{n} \end{array}\right)$$

Let the unit matrix in Equation (11) be $A_{3}$, and f be ${\left(-2x_{k1},-2y_{k1},-2x_{k2},-2y_{k2},\ldots \right)}^{T}$.

In summary, combining Equations (17), (21), and (24), the total cost of path smoothing is

(25) $$cost=x^{T}(\omega_{1}A_{1}^{T}A_{1}+\omega_{2}A_{2}^{T}A_{2}+\omega_{3}A_{3}^{T}A_{3})x+\omega_{3}c^{T}x$$

Rewriting equation (24) into the standard form of quadratic programming, the expression for the Hessian matrix in equation (5) can be written as $H=2(\omega_{1}A_{1}^{T}A_{1}+\omega_{2}A_{2}^{T}A_{2}+\omega_{3}A_{3}^{T}A_{3})$, $c^{T}=\omega_{3}f^{T}$. $x_{k}=(x_{k1},y_{k1},x_{k2},y_{k2},\ldots )$ is a vector composed of the original path point coordinates, and $x=(x_{1},y_{1},x_{2},y_{2},\ldots )$ is a vector composed of the smoothed path point coordinates. In order to maintain the general shape of the planned path, the distance between the smoothed path points and the corresponding original path points should be constrained as follows:

(26) $$|x-x_{k}|\leq dist$$

where dist is the threshold value of the distance of the difference, and Equation (25) is deformed as

(27) $$x_{k}-dist\leq x\leq x_{k}+dist$$

Let $lb=x_{k}-dist$ and $ub=x_{k}+dist$ be the upper and lower constraint boundaries of the optimization variable x, respectively. In addition, due to the characteristics of hybrid A-star planning paths, it is necessary to divide the forward and backward paths and smooth them separately. Therefore, it is necessary to add the equation constraints $x_{i}=x_{ki}$ and $y_{i}=y_{ki}$, where i = 1, 2, n − 1, n. It is guaranteed that the consecutive paths smooth the transition and the positional attitude remains unchanged.

## 4. Speed Planning

The path planned by the hybrid A-star contains information about the position and heading of the path points. To obtain a trajectory that can be used for tracking, speed planning is required on top of the path. Since the proposed method in this paper targets the scenario of low-speed traveling under restricted working conditions, Double S-type speed planning is selected [20].

**Figure 6:**
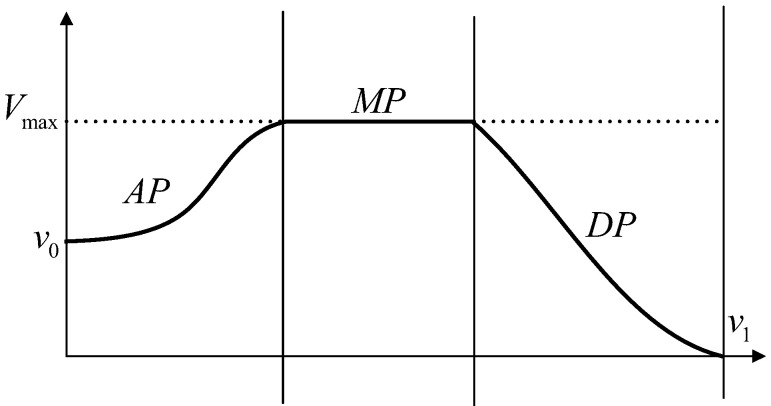
Double S-curve planning speed profile.

Double S-type speed planning solves the problem of acceleration discontinuity by using a combination of two linear segments of intervals. Furthermore, parabolic transitions are used at the endpoints of the linear segments to ensure that the acceleration profile at the connection is continuous. The speed profile of Double S-type speed planning is shown in Figure 6. The overall speed can be divided into three processes, “A”, “M”, and “D”, which are the acceleration phase (AP), maximum speed phase (MP), and deceleration phase (DP). A parabolic fit is used at the endpoints of the AP and DP segments, thus avoiding sudden changes in speed.

Speed planning parameters are constraints on the shape and trend of the speed profile, and different planning effects are realized through different parameter settings. The parameters in Double S-type speed planning can be divided into input parameters and system parameters. The input parameters are displacement at the planning start point, displacement at the planning endpoint, initial speed at the planning start point, and speed at the planning endpoint. The input parameters are set in real-time according to different path lengths to realize different dynamic speed planning. The system parameters are upper and lower speed limits ($v_{max}$, $v_{min}$), limit acceleration ($a_{max}$, $a_{min}$), and limit jerk ($j_{max}$, $j_{min}$). The system parameters depend on the performance requirements and design specifications of the system, and they affect the response speed, stability, and accuracy of the mechanical system.

**Figure 7:**
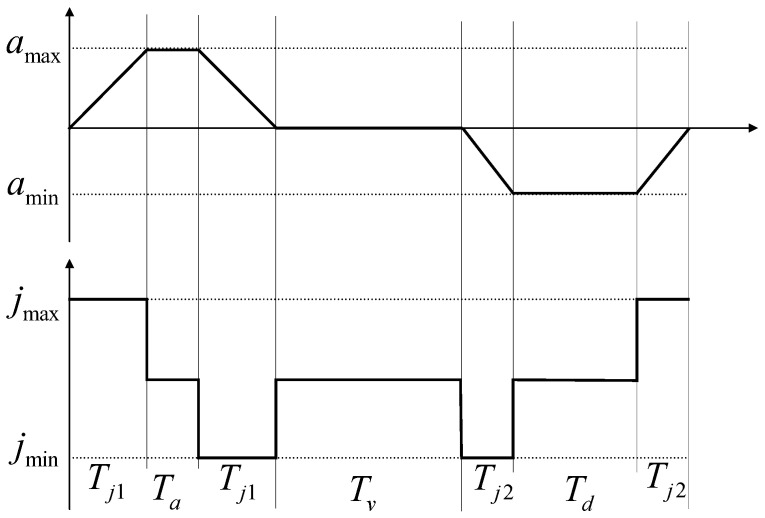
Schematic diagram of speed planning acceleration changes.

It is assumed that $q_{1}>q_{0}$; i.e., the vehicle is in the forward state for the starting and ending displacements. The speed planning problem is now derived based on this assumption. Figure 7 shows the three phases of speed planning acceleration and acceleration changes in different phases of acceleration.

In Figure 7, $T_{j1}$ is the high and low pulse time of the first stage; $T_{j2}$ is the high and low pulse time of the second stage; $T_{a}$ is the time of the acceleration stage $\left(T_{a}\geq 2T_{j1}\right)$; $T_{d}$ is the time of the deceleration stage $\;\left(T_{d}\geq 2T_{j2}\right)$; and $T_{v}$ is the time of the uniform speed stage. In addition, the total planning time $T=T_{a}+T_{d}+T_{v}$. If a constant speed phase exists, the maximum speed of the actual plan $v_{lim}=\max\{\dot{q}(t)\}$ equals the set maximum speed. The actual planned maximum acceleration $a_{\lim}=\max\{\ddot{q}(t)\}$. It is worth noting that not all planning parameters are amenable to Double S-type speed planning. In some limit cases, there is only acceleration or deceleration, i.e., one positive and one negative plus acceleration pulse. The basic condition for planning is to complete at least one “S” curve, and the time taken for a single pulse of the S-curve is $T_{j}^{*}$, which is categorized into two cases depending on whether or not the acceleration reaches the maximum acceleration during the run:

(28) $$T_{j}^{*}=\min\left\{\sqrt{\frac{\left|v_{1}-v_{0}\right|}{j_{max}}},\frac{a_{max}}{j_{m}ax}\right\}$$

$T_{j}^{*}$ is taken as the minimum of the final failure to reach the maximum acceleration and the time to reach the maximum acceleration. Suppose the additive acceleration of the system is considered to be infinite. In that case, i.e., the maximum acceleration can be reached instantaneously at the starting point of planning, then there are the following plannable displacement constraints:

(29) $$q_{1}-q_{0}>\left\{\begin{array}{l} T_{j}^{*}(v_{0}+v_{1})\\ \frac{1}{2}(v_{0}+v_{1})[T_{j}^{*}+\frac{\left|v_{1}-v_{0}\right|}{a_{max}}] \end{array}\right.$$

The ultimate goal of Double S planning is to rationalize the allocation of time among the three phases of A.M.D. to accomplish speed planning.

### 4.1. Calculation of Speed Planning Parameters

The key to accomplishing Double S-type speed planning is the calculation of the parameters, which need to be calculated as the time parameters $T_{j1}$, $T_{j2}$, $T_{a}$, $T_{d}$, and $T_{v}$ and the actual running parameters $v_{\lim}$ and $a_{\lim}$.

#### 4.1.1. Time Parameter

The time parameter calculation is divided into two cases:

(30) $$\left\{\begin{array}{l} v_{lim}=v_{max}\\ v_{lim}<v_{max} \end{array}\right.$$

That is, the existence of a homogeneous phase and the absence of a homogeneous phase throughout.

(1) Assuming that the ($T_{a}$, $T_{d}$) segment exists, first determine whether the accelerated segment reaches the maximum acceleration and then calculate. Calculate the parameters of the segment:

(31) $$(v_{max}-v_{0})j_{max}<a_{max}^{2}$$

If Equation (30) holds, i.e., the maximum acceleration has not been reached, and there is no uniform phase,

(32) $$T_{j1}=\sqrt{\frac{v_{max}-v_{0}}{j_{max}}}$$

(33) $$T_{a}=2T_{j1}$$

If Equation (31) does not hold, i.e., the planning process reaches the maximum acceleration,

(34) $$T_{j1}=\frac{a_{max}}{j_{max}}$$

(35) $$T_{a}=T_{j1}+\frac{v_{max}-v_{0}}{a_{max}}$$

On $T_{d}$ segment parameter calculation,

(36) $$(v_{max}-v_{1})j_{max}<a_{max}^{2}$$

If Equation (36) holds, the maximum deceleration has not been reached, and there is no homogeneous segment:

(37) $$T_{j2}=\sqrt{\frac{v_{max}-v_{1}}{j_{max}}}$$

(38) $$T_{d}=2T_{j2}$$

If Equation (36) does not hold, the maximum deceleration is reached:

(39) $$T_{j2}=\sqrt{\frac{a_{max}}{j_{max}}}$$

(40) $$T_{d}=T_{j2}+\frac{v_{max}-v_{1}}{a_{max}}$$

According to $T=T_{a}+T_{d}+T_{v}$, the uniform time is $T_{v}$.

(41) $$T_{v}=\frac{q_{1}-q_{0}}{v_{max}}-\frac{T_{a}}{2}(1+\frac{v_{0}}{v_{max}})-\frac{T_{d}}{2}(1+\frac{v_{1}}{v_{max}})$$

#### 4.1.2. Actual Operating Parameters

In determining the time, this parameter is divided into a variety of scenarios, so in the system, parameters may change under the current time parameters. To ensure the feasibility of speed planning, it is necessary to modify the desired system parameters: $v_{\max}$ down to $v_{\lim}$, and $a_{\max}$ down to $a_{\lim}$. The procedure for calculating the actual operation of this parameter according to time is as follows:

(42) $$a_{lim_{a}}=j_{max}T_{j1}$$

(43) $$a_{lim_{d}}=j_{max}T_{j2}$$

(44) $$v_{lim}=v_{0}+(T_{a}-T_{j1})a_{lim_{a}}=v_{1}-(T_{d}-T_{j2})a_{lim_{d}}$$

At this time, all parameters used for speed planning are calculated.

### 4.2. Segmented Expression

To ensure a smooth transition of speed and acceleration during motion and to avoid sudden changes in acceleration, segmented calculations using different motion curves for different stages are employed. The acceleration phase (AP) is divided into three stages: increasing acceleration, constant acceleration, and decreasing acceleration. Similarly, the deceleration phase (DP) is divided into three stages: increasing deceleration, constant deceleration, and decreasing deceleration. The solution for each stage is as follows:

1. Increasing Acceleration Phase

At time t∈[0, $T_{j1})$, and acceleration from 0 gradually increased to the maximum value, forming an S-shaped curve half, jerk is positive. The calculation process is as follows:

(45) $$\left\{\begin{array}{l} q(t)=q_{0}+v_{0}t+j_{max}\frac{t^{3}}{6}\\ \dot{q}(t)=v_{0}+j_{max}\frac{t^{2}}{2}\\ \ddot{q}=j_{max}t\\ q^{\left(3\right)}(t)=j_{max} \end{array}\right.$$

2. Constant Acceleration Phase

At the time $t\in [T_{j1}$, $T_{a}-T_{j1})$, after the acceleration reaches its maximum value, the acceleration is kept constant until it needs to be reduced. The calculation process is as follows:

(46) $$\left\{\begin{array}{l} q(t)=q_{0}+v_{0}t+\frac{a_{lim_{a}}}{6}(3t^{2}-3T_{j1}t+T_{j1}^{2})\\ \dot{q}(t)=v_{0}+a_{lim_{a}}(t-\frac{T_{j1}}{2})\\ \ddot{q}=a_{lim_{a}}\\ q^{\left(3\right)}(t)=0 \end{array}\right.$$

3. Decreasing Acceleration Phase

At time $t\in [T_{a}-T_{j1}$, $T_{a})$, in this phase, the acceleration gradually decreases to 0, completing the other half of an S-curve, plus the acceleration is negative. The calculation process is as follows:

(47) $$\left\{\begin{array}{l} q(t)=q_{0}+(v_{lim}+v_{0})\frac{T_{a}}{2}-v_{lim}(T_{a}-t)-j_{lim}\frac{{\left(T_{a}-t\right)}^{3}}{6}\\ \dot{q}(t)=v_{lim}+j_{min}\frac{{\left(T_{a}-t\right)}^{2}}{2}\\ \ddot{q}=-j_{min}(T_{a}-t)\\ q^{\left(3\right)}(t)=j_{min}=-j_{max} \end{array}\right.$$

4. Constant Speed Phase

At the time $t\in [T_{a}$, $T_{a}+T_{v})$, the speed remains constant and does not change anymore. The calculation process is as follows:

(48) $$\left\{\begin{array}{l} q(t)=q_{0}+(v_{lim}+v_{0})\frac{T_{a}}{2+v_{lim}}(t-T_{a})\\ \dot{q}(t)=v_{lim}\\ \ddot{q}=0\\ q^{\left(3\right)}(t)=0 \end{array}\right.$$

5. Increasing Deceleration Phase

After the time $t\in [T-T_{d}$, $T-T_{a}+T_{j2})$, uniform phase, the acceleration gradually increases from 0 to a negative maximum, and jerk is positive, but the speed is decreasing. The calculations are as follows.

(49) $$\left\{\begin{array}{l} q(t)=q_{1}-(v_{lim}+v_{1})\frac{T_{d}}{2}+v_{lim}(t-T+T_{d})-j_{max}\frac{{\left(t-T+T_{d}\right)}^{3}}{6}\\ \dot{q}(t)=v_{lim}-j_{max}\frac{{\left(t-T+T_{d}\right)}^{2}}{2}\\ \ddot{q}=-j_{max}(t-T+T_{d})\\ q^{\left(3\right)}(t)=i_{min}=-j_{max} \end{array}\right.$$

6. Constant Deceleration Phase

At time $t\in [T-T_{d}+T_{j2}$, $T-T_{j2})$, after the acceleration reaches a negative maximum, keep the acceleration constant until the acceleration begins to decrease. The calculation process is as follows:

(50) $$\left\{\begin{array}{l} q(t)=q_{1}-(v_{lim}+v_{1})\frac{T_{d}}{2}+v_{lim}(t-T+T_{d})+\frac{a_{lim_{d}}}{6}[3{\left(t-T+T_{d}\right)}^{2}-3T_{j2}(t-T+T_{d})+T_{j2}^{2}]\\ \dot{q}(t)=v_{lim}+a_{lim_{d}}(t-T+T_{d}-\frac{T_{j2}}{2})\\ \ddot{q}=-j_{max}(t-T+T_{d})\\ q^{\left(3\right)}(t)=0 \end{array}\right.$$

7. Decreasing Deceleration Phase

During time $t\in [T-T_{j2}$, T), the acceleration gradually decreases from the negative maximum value to 0, completing the entire deceleration process. The calculation process is as follows:

(51) $$\left\{\begin{array}{l} q(t)=q_{1}-v_{1}(T-t)-j_{max}\frac{{\left(T-t\right)}^{3}}{6}\\ \dot{q}(t)=v_{1}+j_{max}\frac{{\left(T-t\right)}^{2}}{6}\\ \ddot{q}=-j_{max}(T-t)\\ q^{\left(3\right)}(t)=j_{max} \end{array}\right.$$

The above planning processes are all performed under the premise that $q_{1}>q_{0}$. Now, considering $q_{1}<q_{0}$, this paper proposes a conversion method that makes it unnecessary to distinguish between these two cases when dealing with speed planning, thus simplifying the implementation of the algorithm. Firstly, the initial values are converted as follows:

(52) $$\left\{\begin{array}{l} q_{0}=\Xi \hat{q_{0}}\\ q_{1}=\Xi \hat{q_{1}}\\ v_{0}=\Xi \hat{v_{0}}\\ v_{1}=\Xi \hat{v_{1}} \end{array}\right.$$

where $\Xi =\mathrm{sign}({\hat{q}}_{1}-{\hat{q}}_{0})$ is the sign factor; ${\hat{q}}_{0}$ and ${\hat{q}}_{1}$ are the original input start and end displacements; and ${\hat{v}}_{0}$ and ${\hat{v}}_{1}$ are the original input start and end velocities, respectively. By the above operation, any case can be converted uniformly into the form of ${\hat{q}}_{1}\geq {\hat{q}}_{0}$; after completing the initial value conversion, further convert the planning parameters such as speed, acceleration, and jerk. The specific conversion formulas are as follows:

(53) $$\left\{\begin{array}{l} v_{max}=\frac{\left(\Xi +1\right)}{2}{\hat{v}}_{max}+\frac{\left(\Xi -1\right)}{2}{\hat{v}}_{min}\\ v_{min}=\frac{\left(\Xi +1\right)}{2}{\hat{v}}_{min}+\frac{\left(\Xi -1\right)}{2}{\hat{v}}_{max}\\ a_{max}=\frac{\left(\Xi +1\right)}{2}{\hat{a}}_{max}+\frac{\left(\Xi -1\right)}{2}{\hat{a}}_{min}\\ a_{min}=\frac{\left(\Xi +1\right)}{2}{\hat{a}}_{min}+\frac{\left(\Xi -1\right)}{2}{\hat{a}}_{max}\\ j_{max}=\frac{\left(\Xi +1\right)}{2}{\hat{j}}_{max}+\frac{\left(\Xi -1\right)}{2}{\hat{j}}_{min}\\ j_{min}=\frac{\left(\Xi +1\right)}{2}{\hat{j}}_{min}+\frac{\left(\Xi -1\right)}{2}{\hat{j}}_{max} \end{array}\right.$$

Finally, after obtaining the results of the speed planning, the results need to be converted back to the original directions:

(54) $$\left\{\begin{array}{l} \hat{q}(t)=\Xi q(t)\\ \hat{v}(t)=\Xi \dot{q}(t)\\ \hat{a}(t)=\Xi \ddot{q}(t)\\ \hat{j}(t)=\Xi q^{\left(3\right)}(T) \end{array}\right.$$

Through the conversion of the above steps, different situations in speed planning can be handled uniformly, making the algorithm more concise and general. This not only improves the computational efficiency but also ensures the smooth transition and stable operation of the system under different initial conditions. The method of unified initial value and planning parameter conversion provides an effective solution for dealing with complex motion control problems.

### 4.3. Parameter Settings

Since the trajectory planned by the hybrid A-star algorithm includes both forward and backward paths, segmented planning is required, and the speed at the endpoint of each path segment should be zero. If a path segment is too short, i.e., $\left|q_{0}q_{1}\right|$ is smaller than a certain threshold, $T_{v}=0$ may arise. To ensure the ride comfort of the autonomous vehicle and to avoid mechanical loss caused by rapid acceleration and deceleration, a mechanism is designed to dynamically adjust system parameters according to the path length. This ensures that the speed planning protocol always includes a constant speed phase. First, the path curve length is calculated:

(55) $$L=\sum_{i=1}^{n-1}\sqrt{{\left(x_{i+1}-x_{i}\right)}^{2}+{\left(y_{i+1}-y_{i}\right)}^{2}}$$

where L is the curve passing length of each path segment; n is the number of discrete points of the path. The system parameters $q_{0}=0$, $q_{1}=L$ (vehicle in forward direction), $q_{0}=L$, $q_{1}=0$ (vehicle in backward direction), and $v_{0}=v_{1}=0$ are set according to the dynamic path length. The system parameters maximum speed $v_{\max}$ and maximum acceleration $a_{\max}$ are dynamically adjusted as follows:

(56) $$\left\{\begin{array}{l} v_{max}=v_{base}+k_{v}L\\ a_{max}=a_{base}+k_{a}L \end{array}\right.$$

where $v_{base}$ is the system base speed, $k_{v}$ is the speed gain coefficient, $a_{base}$ is the system base acceleration, and $k_{a}$ is the acceleration gain coefficient. Both $v_{base}$ and $a_{base}$ are set according to the system’s minimum acceleration requirements. Additionally, the system parameters in this paper are set symmetrically as follows:

(57) $$\left\{\begin{array}{l} j_{min}=-i_{max}\\ a_{min}=-a_{max}\\ v_{min}=-v_{max} \end{array}\right.$$

Since Double S planning is one-dimensional, the speed and acceleration need to be discretized according to the number of discrete path points. The final mapping to the discrete points of the planned path results in a trackable trajectory containing position, attitude, speed, and acceleration information.

## 5. Simulation and Experimentation

To verify the effectiveness of the method proposed in this paper in a constrained space, simulation and experimental validation are carried out using the Robot Operating System (ROS).

### 5.1. Simulation Verification

#### 5.1.1. Simulation Platform

**Figure 8:**
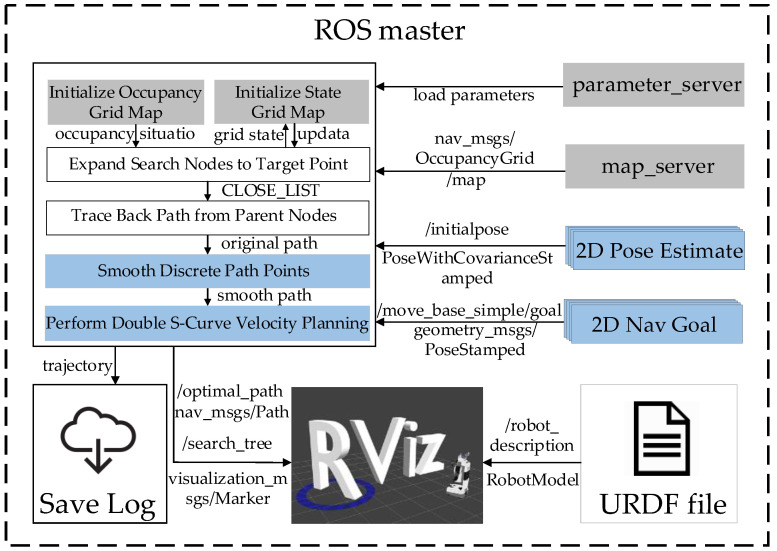
Schematic diagram of simulation flow.

The operating environment consists of a 64-bit Linux operating system, Ubuntu 20.04, with ROS1 Noetic. The simulation flow is illustrated in Figure 8. First, load the map information using the built-in “map_server” node in the ROS system. Read the algorithm parameters from the parameter manager. Use “2D Pose Estimate” and “2D Nav Goal” to set the initial and endpoints’ positions and orientations. Initialize the state grid and raster map based on the map, initial point, and endpoint information. Node management is handled through two lists, OPEN_LIST and CLOSE_LIST, which are used to backtrack each node at the end of the search to reconstruct the original path. Perform speed planning on the smoothed path to generate a trackable trajectory. Publish the planned trajectory and the search tree generated during the search. Additionally, read the URDF file and publish the vehicle model as a topic. Finally, visualize the planned trajectory, search tree, and vehicle model in Rviz. The algorithm’s planning results are saved in real-time using file input and output streams in C++17.

Two scenarios were selected for validation: Scene 1—continuous regular obstacle scenario. Scene 2—discrete irregular obstacle scene. In each scene, the same starting point and endpoint are set, and the method of this paper (IHAS) is compared with the traditional A-star (THAS) algorithm. Combining the parameters of the simulated vehicles and the test scenes in this paper, the parameters of the hybrid A-star search-related algorithm are set as $\Delta s_{0}=0.2;\Delta \theta_{0}=30;\alpha =0.52;\beta =0.5;\Delta s_{\max}=0.2;\Delta \theta_{\max}=0.8$. The resolution of the grid map is 0.2 (i.e., each grid cell represents 0.2 m). Path smoothing algorithm parameters are set as $\omega_{1}=100;\omega_{2}=5;\omega_{3}=5.5$. Speed planning this parameter is set as Ξ=0.7; $v_{base}=10;a_{base}=15;k_{v}=0.5;k_{a}=0.5$. In addition, to ensure the fairness of the comparison, the parameters of the three methods are set to the same parameters.

#### 5.1.2. First Scenario

**Figure 9:**
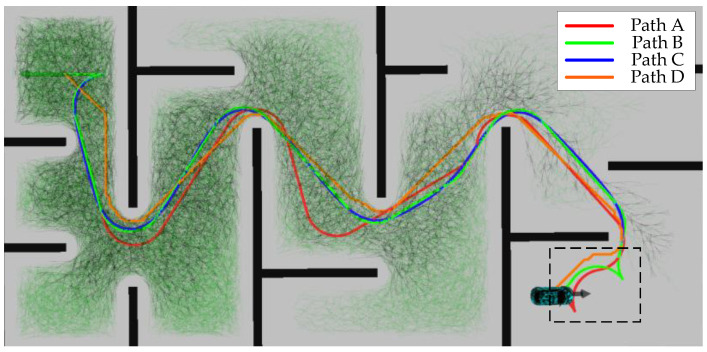
Path planning results within the first scenario.

The first scenario aims to simulate the extreme working conditions of narrow passages and long straight walls. In turn, it evaluates the ability of the algorithms proposed in this paper in terms of path smoothing, handling vehicle kinematics constraints, and search efficiency. The trajectory planning results of different methods in the first scenario are shown in Figure 9.

The specific meanings of the four paths in Figure 9 are Path A represents the planning result of the traditional hybrid A-star algorithm; Path B represents the planning result of the hybrid A-star algorithm with the improvement in the node expansion method and the estimation of the cost h(*n*) in this paper; Path C is the result of smoothing Path B through the back-end smoothing method designed in this paper; and Path D is the shortest path searched for between the start point and the endpoint by using Dijkstra’s algorithm. From Figure 8, it can be observed that compared with Path A, the curvature change in Path B is more continuous, and there is no steering. Since the hybrid A-star method can perform path planning while ensuring the vehicle’s kinematics, it also addresses the vehicle’s traveling states, including both forward and reverse states. In the method designed in this paper, the RS curve connects the current position with the target position when approaching the target point. RS curve planning maintains the vehicle’s kinematics and considers the vehicle’s traveling state. The primary goal of designing the ‘spike’ shape of the path in the black dashed area in Figure 9 is to accommodate the reversing maneuver. Planning the reversing state optimizes the vehicle’s trajectory to adjust the path effectively without compromising kinematic performance. This approach enhances the vehicle’s maneuverability and operational flexibility in confined spaces, ensuring that path planning meets kinematic constraints while adapting to actual driving needs. Moreover, the overall trend of Path B is closer to the trend of the shortest path, Path D. In addition, the improved hybrid A-star algorithm produces a significantly smaller size of the number of searches. The time consumed by the two methods to complete the search is shown in Table 1: 

The data in Table 1 show that the time consumed is relatively high due to the relative complexity of the improved computation of the cost h(*n*). However, the time from collision detection and obtaining neighboring nodes is substantially reduced. There are two main reasons for this: on the one hand, it is because the node expansion method adopts a variable step size, which reduces the number of sampled nodes; on the other hand, the cost of expanding nodes is evaluated more accurately. Thus, sacrificing the time for calculating the cost leads to a 73.03% reduction in the total used time. In addition to this, the number of discrete points for planning and the path length for searching are reduced by 12.13% and 6.25%, respectively.

**Remark 2.**  To verify the effectiveness of the method proposed in this paper in a constrained space, the simulation time consumed by the hybrid A* approach to planning includes the following main components: computing the cost of a node, extending neighboring nodes, and detecting collisions. Dijkstra’s shortest path algorithm provides the optimal direction for expanding nodes, which avoids extensive searches and reduces the time consumed. Additionally, this approach minimizes redundant nodes in the search process, thereby reducing the collision detection time for these redundant nodes.

**Figure 10:**
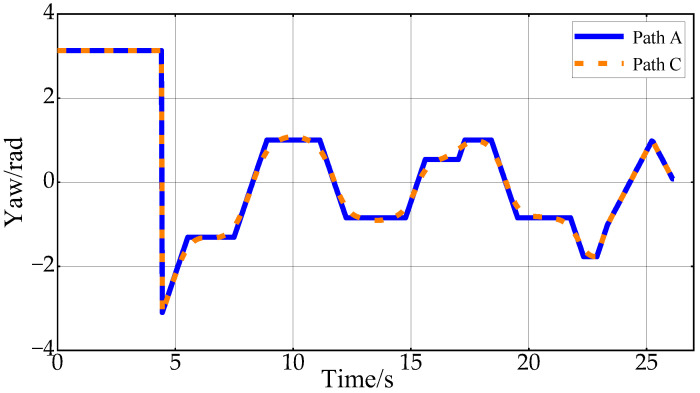
Vehicle heading change in the first scenario.

Figure 10 shows the desired vehicle heading angle changes corresponding to Path B and Path C. In this paper, the vehicle heading angle in the range of 0–2π continues to increase at 4.3 s. Thus, the vehicle heading angle remains continuously variable. The Reeds–Shepp curve is used to connect the endpoint when approaching the target point. In order to ensure that the position of the planning endpoint is consistent with the desired goal point, the back-end smoothing of the planning path in this paper does not include the RS path. From the figure, it can be concluded that the smoothed path expects smoother heading angle changes, which improves the ride comfort of the vehicle in the lateral direction.

**Figure 11:**
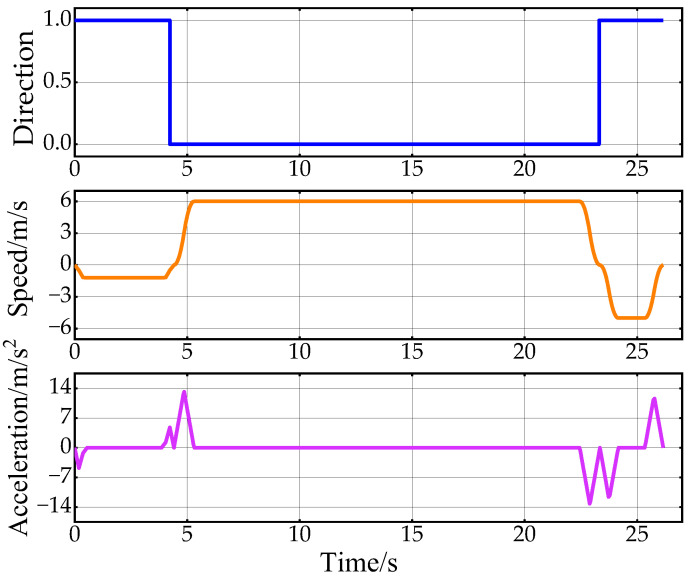
Speed planning results for the first scenario.

Figure 11 shows the results of the speed planning for Path C. It depicts the direction of travel of the vehicle in different phases as well as the planned speed and acceleration. If the value of “Direction” is 1, it means the vehicle state is forward; otherwise, the vehicle state is backward. Due to the initial position and environmental constraints, the vehicle heading is adjusted by reversing at the beginning of the phase. From the figure, it can be seen that the planned speed and acceleration directions are consistent with the vehicle state, and the changes are continuous. The trend of the speed and acceleration can be seen through the change in speed and acceleration, the adaptive adjustment system parameter mechanism designed in this paper to limit the speed and acceleration for short distances.

#### 5.1.3. Second Scenario

The location and shape of the obstacles in the second scenario are irregular. The adaptability and generalization ability of the algorithms in this paper in real application environments can be evaluated to ensure their effectiveness in various complex situations. The planning results of different methods under Scene 2 are shown in Figure 11.

**Figure 12:**
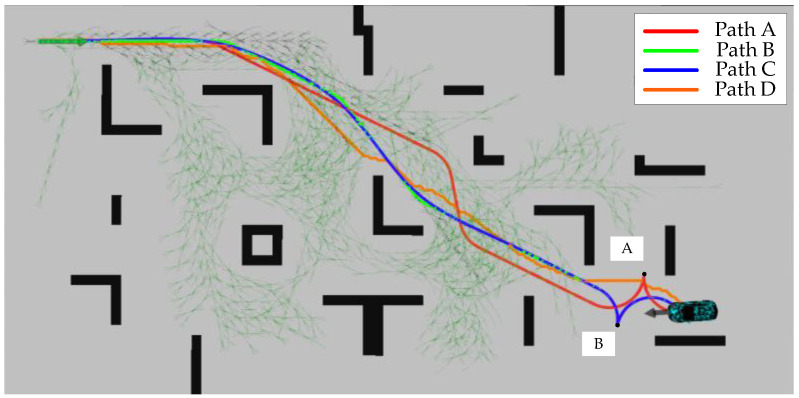
Path planning results within the second scenario.

Path A, Path B, Path C, Path D, and other elements in Figure 12 have the same meaning as in the first scenario. The most obvious change compared to scenario one is that the search tree for both methods, IHAS and THAS, is significantly reduced. This is due to the fact that in irregular obstacle scenarios, the random distribution of obstacles leads to an increase in the diversity of path choices. The hybrid A-star algorithm can utilize more free space to generate shorter and straighter paths, thus reducing the number of node expansions. Whereas in the scenario with regular obstacles (e.g., long passages), there are fewer path choices and the algorithm may need to explore more nodes to find the only feasible path, increasing the number of search trees. However, in the second scenario, the search tree size of IHAS is still significantly smaller than that produced by THAS. It is more obvious in the second scenario that the paths planned by IHAS are closer to the shortest path, Path D, while satisfying the vehicle kinematics. Points A and B in Figure 12 represent the forward and reverse state thresholds in the paths planned by the THAS and IHAS methods, respectively. This approach ensures that the final position and attitude planned along the shortest path align with the desired state. In addition, the time consumed to complete the search by the two methods is shown in Table 2.

As can be seen from Table 2, all the used times of IHAS in the second scenario are less than the THAS method. For the efficiency of searching a path, the IHAS method improves 88.69% relative to the HAS method. This result clashes with the size of the search tree it produces. It is worth noting that the excessive redundant nodes searched by the THAS method lead to an overall decrease in the used time to compute h(*n*), even for the IHAS method, which has a more complex estimated cost. The node expansion approach with variable step size and angle and improved estimated cost resulted in an 8.23% reduction in the number of planned path points and a 2 m reduction in the total path length.

**Figure 13:**
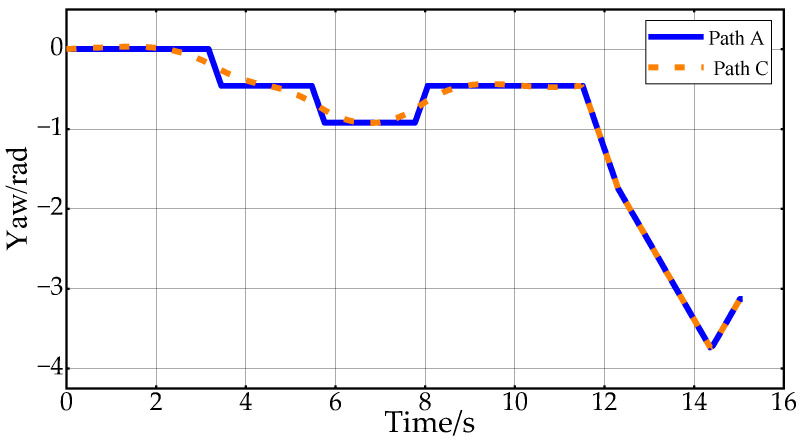
Vehicle heading change in the second scenario.

Figure 13 shows the variation of desired heading angles for Path C and Path D within the second scenario. In complex environments, the smoothed path heading angle changes more gently, making the path more feasible and comfortable.

**Figure 14:**
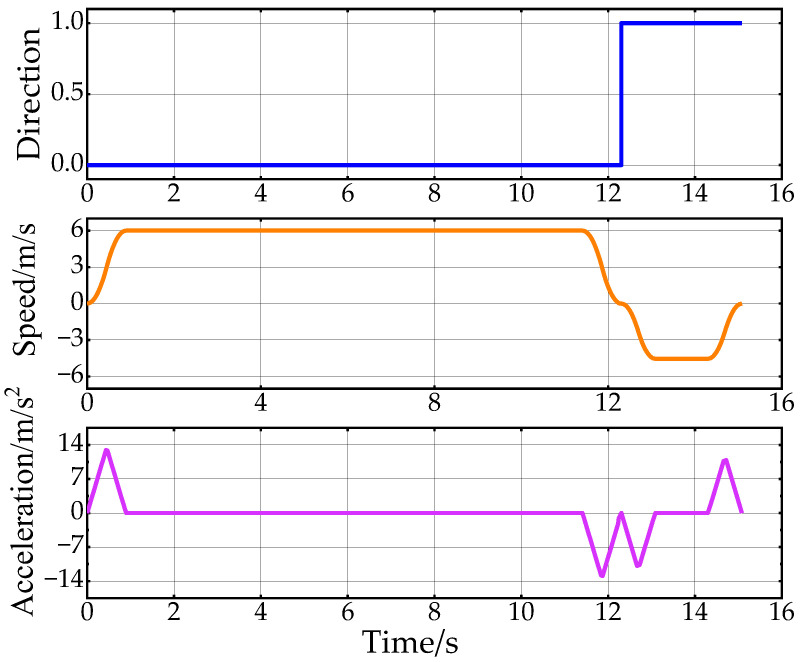
Speed planning results for the second scenario.

Figure 14 shows the results of the speed planning within the second scenario. Due to the settings of the start and end poses, the whole journey is divided into two segments for speed planning. In the first 12.5 s, the vehicle is moving forward, accelerates to 6 m/s, and then starts to decelerate at 12 s. After decelerating to 0, the vehicle starts to reverse, and after the uniform speed phase, it starts to decelerate to 0. Finally, the vehicle is parked at the target point with the desired attitude. The speed change process is continuous and smooth to ensure the ride comfort of the vehicle in the longitudinal direction.

#### 5.1.4. Third Scenario

**Figure 15:**
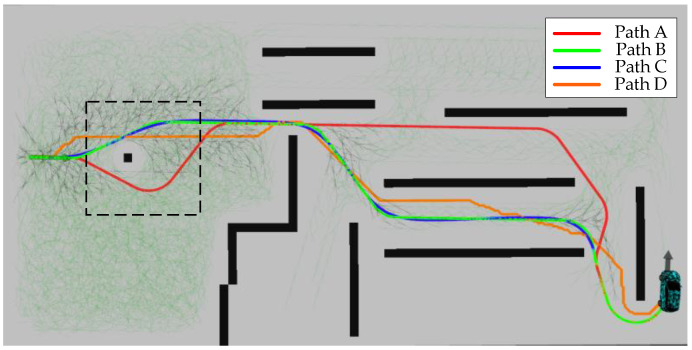
Path planning results within the third scenario.

In this paper, a dynamic expansion of nodes based on the complexity of the environment and the distance to the target point is designed. This method improves the algorithm’s efficiency, but it may decrease obstacle avoidance ability in low-complexity environments because the step size is too large. In order to test the performance of the proposed method in this paper in terms of obstacle avoidance ability in low-complexity environments and planning under narrow space, a third scenario is designed, as shown in Figure 15.

Path A, Path B, Path C, Path D, and other elements in Figure 15 have the same meaning as in the first scene. The dashed box in the figure shows the area far from the target point and has relatively low environmental complexity. The planning results show that obstacle avoidance planning can still be completed in the scene with a relatively large step size. Although the shortest path, Path D, violates the vehicle kinematics constraints, Path D provides a guideline of the shortest path for the IHAS method in a narrow channel. The paths planned by THAS are shorter compared to the traditional method THAS. With Table 3, all the elapsed times of the IHAS method are improved. It is because of the correct guidance of the shortest path, avoiding ‘more detours’ in the search process. This is confirmed by the distribution of the search tree in Figure 15.

**Figure 16:**
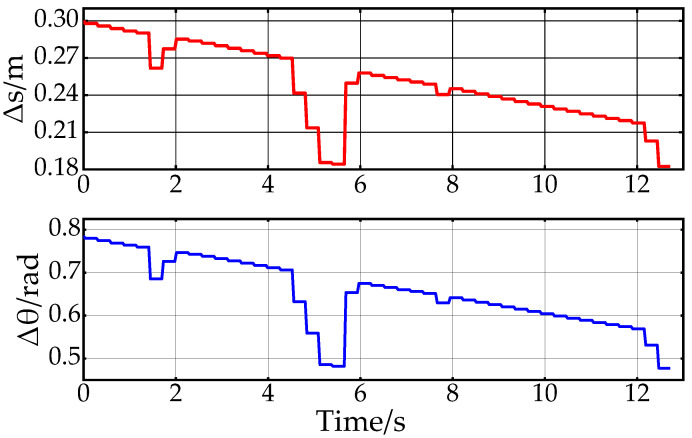
Dynamic extension of step size variation in the third scenario.

Figure 16 shows the dynamic expansion step change in the third scenario. As can be seen from the figure, the distance step size Δs and the angle step size Δθ dynamically change during the search. As the distance to the target point gets closer, the overall trend of the step length gradually decreases. At the same time, the magnitude of the dynamic step length is adjusted in real-time with the changes in environmental complexity. Combined with the time consumption of the algorithm in Table 3, it further illustrates the effectiveness of the step length dynamic expansion strategy designed in this paper. In addition, the path smoothing and speed planning results under the third scenario have the same effect as those under the first and second scenarios.

### 5.2. Real-Vehicle Experiment

**Figure 17:**
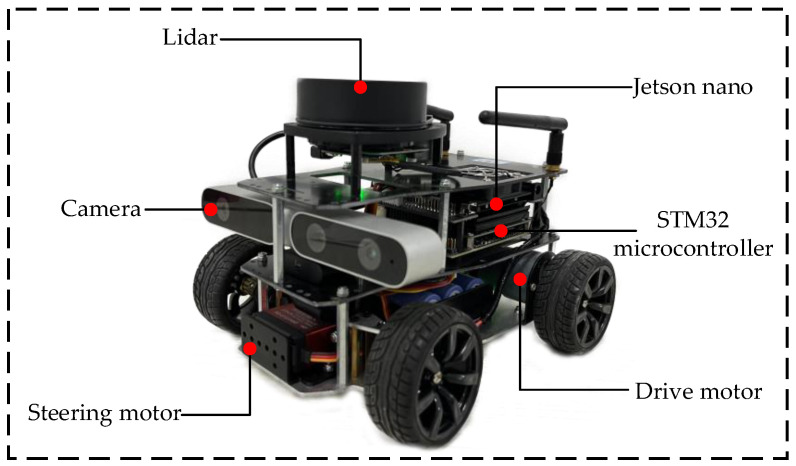
Diagram of ROS smart experimental vehicle.

**Figure 18:**
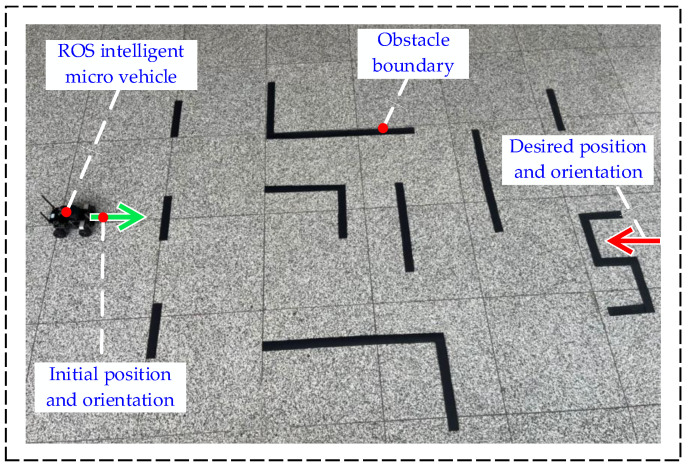
Schematic diagram of experimental scene.

Real-vehicle experiments can be used to validate the effectiveness of the method proposed in this paper in a natural environment based on simulation. The hybrid A-star and trajectory smoothing parameters are set as in the simulation, and the velocity planning parameters are set as follows: Ξ=0.7; $v_{base}=6;a_{base}=10;k_{v}=0.5;k_{a}=0.5$. The object of the experiment is the ROS intelligent micro-vehicle, as shown in Figure 17. The experimental scene was built manually, as shown in Figure 18.

#### 5.2.1. Experimental Platforms

The traveling state of the micro-vehicle in Figure 17 supports forward and backward motion and satisfies the vehicle kinematics constraints. The experimental trolley is equipped with a single-line LiDAR (RPLIDARA1360) to detect the position of static. A depth camera (IMX219-160) handles target detection and provides category and depth information. The STM32 microcontroller, embedded with an Inertial Measurement Unit (MPU9250), delivers linear and angular acceleration data in three directions, which helps in calculating real-time vehicle speed and position. The computing platform (Jetson Nano) processes complex perception, decision-making, and control algorithms, extracting sensor data for target recognition, path planning, and motion control. The high-performance computing and deep learning capabilities of Jetson Nano enable the intelligent micro-vehicle to respond more quickly and accurately to environmental changes.

In Figure 18, the ROS trolley is used as the controlled object. The green arrow is the initial position, and the red arrow is the desired position and orientation of the target point. The controlled vehicle needs the green arrow to depart to reach the red arrow, and the orientation is the same as that of the red arrow. Due to the limitation of the experimental site and the trolley’s structural characteristics, the experiments are carried out under low-speed conditions.

#### 5.2.2. Analysis of Experimental Results

**Figure 19:**
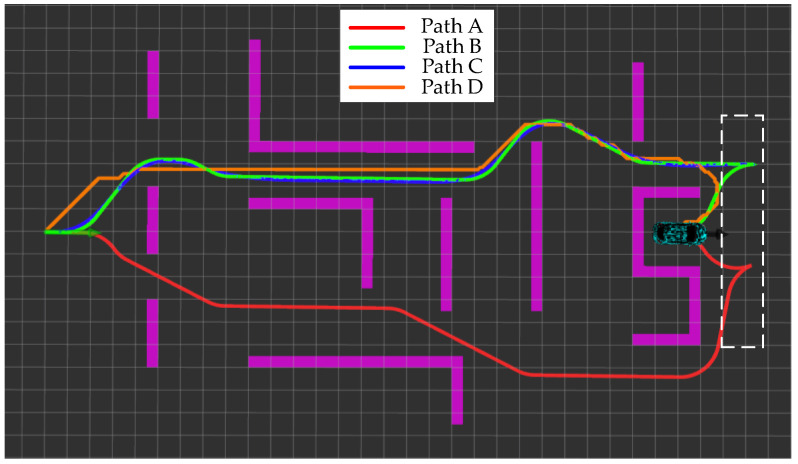
Results of experimental planning with real vehicle.

Figure 19 shows the results of the real-vehicle experiment. The algorithm performs path planning by providing an a priori map of the cart. Paths A, B, C, and D are THAS planned paths, IHAS planned paths before smoothing, IHAS planned paths, and Dijkstra planned paths, respectively. In the experiment, the path followed by the controlled vehicle is Path B. Due to the characteristics of obstacles, start position, and target position, Path D is very different from Path A. The path planned by IHAS is shorter than that planned by THAS under the guidelines of the shortest path. Due to the characteristics of the desired attitude, both IHAS and THAS, there is a reversing maneuver in the final stage to achieve the desired attitude. The paths planned by the IHAS and THAS methods consume 68 ms and 145 ms, respectively, with an overall improvement in search efficiency of 53.13%.

**Figure 20:**
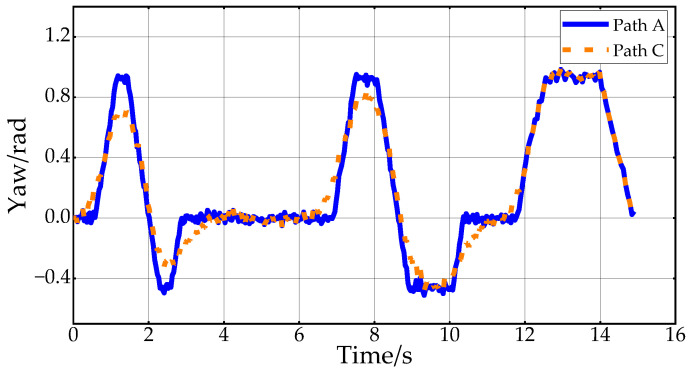
Vehicle heading changes in the experiment.

**Figure 21:**
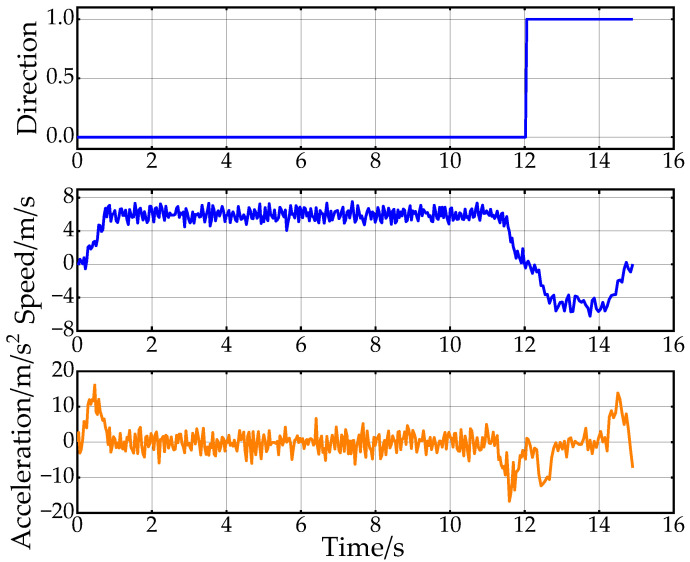
Speed planning results from real-vehicle experiments.

Figure 20 shows the changes in vehicle heading during the experiment. The heading information is obtained by integrating the angular velocity measurements from the onboard IMU sensor. Due to the vehicle’s mechanical structure and the sensor’s characteristics, the collected heading information is noisy. However, the overall trend in Path A and Path C indicates that the heading change in Path C is smoother, further proving the effectiveness of the proposed back-end smoothing method. Figure 21 displays the results of the experimental speed planning for the real vehicle. Acceleration data are directly obtained from the IMU’s linear acceleration measurements, and velocity is derived by integrating this acceleration. The overall velocity change is smooth, with the velocity decreasing to zero at 12 s before entering a reverse state with negative velocity. These results further validate the rationality and effectiveness of applying Double S velocity planning to hybrid A-star planned paths.

Combining the above analyses, the method proposed in this paper demonstrates effective performance in real-world constrained environments. It successfully plans the shortest safe path while adhering to the vehicle’s kinematic constraints. Additionally, path and speed smoothing further enhances the vehicle’s ride comfort.

## 6. Conclusions

In this paper, an optimal trajectory planning method under restricted narrow space is proposed. The traditional hybrid A-star algorithm is improved based on the traditional hybrid A-star algorithm, and the search capability is improved by more than 50% in different scenarios. At the same time, back-end smoothing is performed on the searched paths. The improved hybrid A-star algorithm has higher adaptability and robustness in dealing with complex continuous obstacles and irregular obstacles. The simulation analysis and real-vehicle experiment show that the improved algorithm in this paper performs well in a variety of complex scenarios, which not only significantly reduces the computation time of path planning but also significantly improves the smoothness and safety of the path. In addition, the introduction of speed planning further optimizes the motion trajectory of the autonomous vehicle so that it can adaptively adjust the driving speed under different working conditions to improve overall driving efficiency and ride comfort. After the processing of path planning and speed planning, an optimized trajectory is obtained with the comprehensive consideration of path smoothness and speed control.

It is worth noting that the method proposed in this paper relies on a raster search class algorithm. Although the method improves in some aspects, it still demands significant memory and time. Consequently, this method is more suitable for fixed scenarios like car parks, warehouses, and other limited-area environments. Additional optimization and adaptation may be necessary for large-scale or dynamic environments (e.g., urban planning). Future work will explore more efficient algorithms and optimization strategies to enhance applicability in complex and dynamic environments.

## Figures and Tables

**Table 1:** The time consumption of the search within the first scenario.

	THAS	IHAS
Time to compute cost h(*n*)/ms	27.79	35.89
Time to check for collision/ms	617.56	146.23
Time to obtain neighboring nodes/ms	731.25	174.39
Total search time/ms	816.30	220.25

**Table 2:** The time consumption of the search within the second scenario.

	THAS	IHAS
Time to compute cost h(*n*)/ms	0.99	0.41
Time to check for collision/ms	19.57	1.94
Time to obtain neighboring nodes/ms	23.23	2.32
Total search time/ms	25.65	2.90

**Table 3:** The time consumption of the search within the third scenario.

	THAS	IHAS
Time to compute cost h(*n*)/ms	1.29	0.92
Time to check for collision/ms	21.36	3.91
Time to obtain neighboring nodes/ms	33.23	4.46
Total search time/ms	22.12	2.14

## Data Availability

The data used to support the findings of this study are available from the corresponding author upon request.
